# Effects of Juvenile Host Density and Food Availability on Adult Immune Response, Parasite Resistance and Virulence in a *Daphnia-*Parasite System

**DOI:** 10.1371/journal.pone.0094569

**Published:** 2014-04-15

**Authors:** Corine N. Schoebel, Stuart K. J. R. Auld, Piet Spaak, Tom J. Little

**Affiliations:** 1 Department of Biodiversity and Conservation Biology, Swiss Federal Research Institute WSL, Birmensdorf, Switzerland; 2 Eawag, Dübendorf, Switzerland; and Institute of Integrative Biology, ETH, Zürich, Switzerland; 3 Institute of Evolutionary Biology, University of Edinburgh, Edinburgh, United Kingdom; 4 School of Natural Sciences, University of Stirling, Stirling, United Kingdom; Emory University, United States of America

## Abstract

Host density can increase infection rates and reduce host fitness as increasing population density enhances the risk of becoming infected either through increased encounter rate or because host condition may decline. Conceivably, potential hosts could take high host density as a cue to up-regulate their defence systems. However, as host density usually covaries with food availability, it is difficult to examine the importance of host density in isolation. Thus, we performed two full-factorial experiments that varied juvenile densities of *Daphnia magna* (a freshwater crustacean) and food availability independently. We also included a simulated high-density treatment, where juvenile experimental animals were kept in filtered media that previously maintained *Daphnia* at high-density. Upon reaching adulthood, we exposed the *Daphnia* to their sterilizing bacterial parasite, *Pasteuria ramosa,* and examined how the juvenile treatments influenced the likelihood and severity of infection (Experiment I) and host immune investment (Experiment II). Neither juvenile density nor food treatments affected the likelihood of infection; however, well-fed hosts that were well-fed as juveniles produced more offspring prior to sterilization than their less well-fed counterparts. By contrast, parasite growth was independent of host juvenile resources or host density. Parasite-exposed hosts had a greater number of circulating haemocytes than controls (*i.e.,* there was a cellular immune response), but the magnitude of immune response was not mediated by food availability or host density. These results suggest that density dependent effects on disease arise primarily through correlated changes in food availability: low food could limit parasitism and potentially curtail epidemics by reducing both the host’s and parasite’s reproduction as both depend on the same food.

## Introduction

Host fitness decline due to parasitism (often termed virulence) is commonly context dependent [1]–[4]. For example, recent theoretical studies suggest that the expression of virulence depends on host population density, such that infected hosts have a higher sensitivity to density, and hence reach their carrying capacity earlier than uninfected hosts [5], [6]. Moreover, since increased host density is thought to enhance the potential of parasite transmission [4], [7], elevated juvenile host densities may predict increased likelihood of infection at the adult stage, and thus act as a cue for hosts to shift investment into immune defences. Yet, increased immune preparedness can potentially come with costs - either energetic cost of investment or immunopathological cost when responses are launched [8]. Indeed, immune functions have been shown to trade off with other life history traits. For example, survival was reduced in bumblebees and beetles with challenged immune systems [9], [10], and in Indian meal moths and sticklebacks, an increase in resistance was correlated with longer development time [11], [12].

Changes in host density are likely to be accompanied by changes in food availability and hence host condition (i.e. fitness). This is termed negative density dependence, and is commonly observed in animal and plant populations [13], [14], [15]. Moreover, the incidence and severity of parasitism is often highest when the host is under stressful conditions, for example very low food conditions. This was discussed for the *Daphnia galeata* – *Caullerya mesnili* host-parasite system in a Swiss lake, where infection levels peak in autumn, when host density is still high, but food level is low [16]. An increase in parasite-induced effects under food stress has also been shown in other systems (e.g. parasite specific mortality rates under starvation in snail hosts infected with either of two different micorparasites [17]), and this may partly be explained by the difficulty of maintaining energetically expensive immune functions under low food conditions [18], [19]. However, for butterfly larvae immune parameters were negatively affected by high-density, while starvation did not have any effect [20] and in females of a parthenogenetic freshwater snail, a reduction in reproduction and growth (key fitness traits) was detected under high-density and constant food conditions, compared to low-density [21].

Varying the environmental quality experienced by juvenile hosts may shed light on investment in immune defences and patterns of parasitism in adult hosts. But to what extent is variation in juvenile host condition driven by host density itself (as opposed to correlated effects of food availability)? Progress in understanding the consequences of changing density for parasitism will be aided by experimental designs that simultaneously and independently study variation in host density and variation in host condition (as determined by food). This is a challenging task, because these two factors are normally not independent in natural and experimental systems. Therefore, a treatment of simulated high-density (SHD) could help to disentangle the effects of actual crowding and sensed crowding onto host condition and immunity. For example, for aquatic hosts, a SHD treatment could involve maintaining low densities of hosts in filtered media that previously contained large populations of hosts [22], [23]. In aquatic systems, SHD treatments allow hosts to sense and release waterborne chemical cues without actual physical crowding [24]. However, SHD treatments are not feasible for many model systems and, to our knowledge no study has achieved this goal. We use this experimental approach with a natural host-parasite system: *Daphnia magna* and its bacterial parasite *Pasteuria ramosa*, because previous studies have shown that *Daphnia* are able to sense and react to chemical cues dissolved in the surrounding water that indicate crowding [22]. We present the results of two cross-factored experiment with four different food and three density treatments, including low, high and simulated high-density (a total of 12 treatments). In the first experiment, we record the proportion of hosts that suffer infection following parasite exposure during the adult stage, as well as measures of host fitness in healthy and parasite-infected hosts. In the second experiment, we record immune investment (haemocyte number) in control and parasite-exposed adult hosts.

## Materials and Methods

We used the cyclically parthenogenetic freshwater crustacean *D. magna* and its parasite, the spore-forming bacterium *P. ramosa,* an obligate endoparasite that infects via horizontal transmission of mature spores from dead hosts by ingestion of spores [25]. Successful infection can be easily seen by eye, since *Pasteuria* causes gigantism, red colouration and obvious bacterial growth in the haemolymph of the *Daphnia* (symptoms are visible 8–25 days post-exposure). Infection severely curtails host fitness, with hosts typically becoming completely sterile [25]. We used a single genotype of *D. magna* (GG4) and a single parasite isolate (Sp1). Both the host genotype and parasite isolate were collected in 1997 from a pond near Gaarzefeld, Northern Germany and maintained in laboratory populations ever since (first studied by [26]). Sampling permission was not required, as neither *D. magna,* nor *P. ramosa* are protected or endangered in Germany.

We performed two separate experiments: *Experiment I* examined how the probability of infection and the virulence (defined here as the reduction in host fecundity due to infection) were affected by juvenile food and density conditions (following a fully-factorial design); *Experiment II* was identical in setup, but was dedicated to testing how juvenile food and density conditions affected the numbers of circulating haemocytes in parasite-exposed and non-exposed hosts [27], [28]. Throughout the experiments, *Daphnia* were kept in artificial *Daphnia* media (ADaM [29]) in a 20°C incubator with a 12∶12 hour light: dark cycle. They were fed daily with 1.0 absorbance (abs) of chemostat-grown *Chlorella vulgaris* per *Daphnia* (one abs is the optical absorbance of 650 nm white light by the algae culture). Prior to the experiments, independent host replicates were maintained for three generations in order to minimize variation in maternal effects. *Daphnia* were kept under standard conditions in groups of 5 animals in 200 ml of media. Three times per week they were transferred to fresh media and any offspring were discarded. Second-clutch neonates from the third generation were used to set up the experimental units. The experimental treatments consisted of four pre-exposure food treatments - from excess food - 2 abs, down to low food conditions at 0.25 abs, with intermediate levels of 0.5 and 1.0 abs algae per day and *Daphnia*.

In addition, hosts were kept under three different density pre-exposure treatments: high-density (HD), with 15 *Daphnia* in a 200 ml jar of fresh media, low-density (LD), with 5 *Daphnia* in 200 ml of fresh media and simulated high-density (SHD), with 5 *Daphnia* in 200 ml of filtered media that previously maintained 15 *Daphnia.* The SHD media was filtered through pore size 45 µm inert filters (Sartoban 300, Sartorius Stedim Biotech, Germany) to remove particles such as excess food or moulted *Daphnia* carapaces. In order to collect enough SHD media, we recycled and filtered water from all HD replicates (except the ones fed 2.0 abs of food) as well as additional SHD “water factories” consisting of age matched *Daphnia* kept in HD conditions outside the experiment. We measured host age at first reproduction and size of first clutch to assess the effects of food and density conditions during the juvenile stage.

Parasite exposure was carried out in an identical way for both experiments. Each jar had its own day of maturity. On the day *Daphnia* within a jar reached maturity (defined as the day that over half of the *Daphnia* in each jar had deposited eggs into their brood chamber), five *Daphnia* (all *Daphnia* from the LD and SHD jars and five randomly-chosen *Daphnia* from the HD jars) were exposed to the parasite treatment for five hours as follows. Five *Daphnia* of each replicate-treatment combination were placed in one well of a 24 well cell plate (Costar, Corning Inc., NY) containing 1 ml of media. Parasite-exposed replicates received 50 000 *P. ramosa* spores, a dose commonly used in *D. magna* experiments [26]–[28], [30], [31]. Non-exposed control replicates did not receive any spores, but were placed in a cell plate well for the same amount of time. This resulted in 12 (8 for *Experiment II*) replicates per food, per parasite, and per density treatment, leading to 288 (*Experiment I*) and 192 (*Experiment II*) experimental units (i.e. jars). Permits are not required in order to conduct laboratory experiments with *D. magna* and *P. ramosa*.

### Experiment I

After parasite exposure, a single randomly-chosen *Daphnia* per replicate was kept in an individual jar each containing 60 ml of fresh media and fed 1 abs of *C. vulgaris* per day; the others were discarded. Throughout the experiment, jars were randomly distributed within trays of 24, and tray position within the incubator was randomized daily to reduce the impact of any positional effects. Jars were checked daily for offspring and mortality. If a female had a clutch, the offspring were counted, and the mother (experimental individual) was placed into fresh media. Media was changed every 3 days regardless of whether or not the female had a clutch. On day 25 post-exposure, the proportion of *Daphnia* that had become infected was recorded.

**Figure 1:**
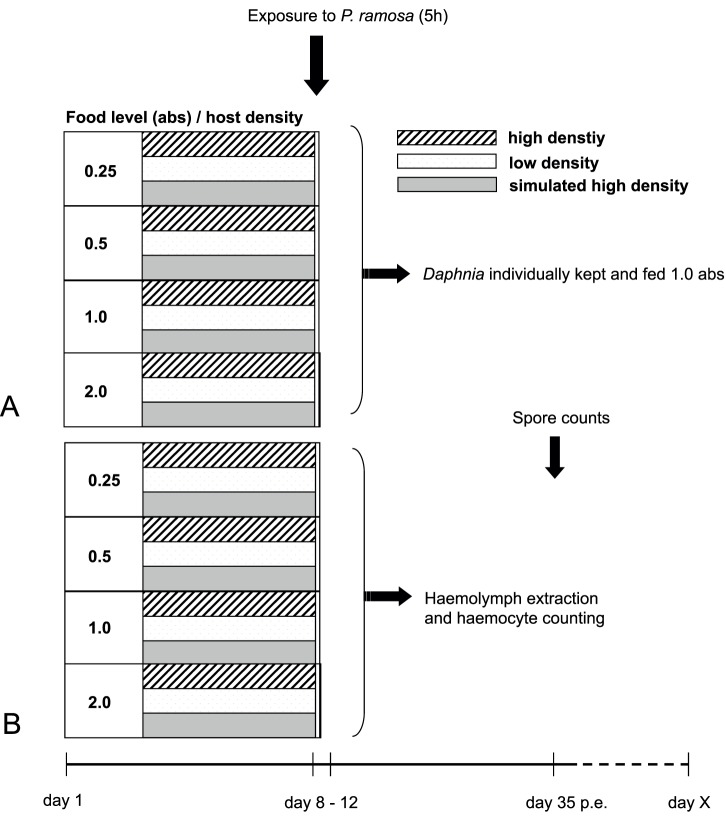
Schematic overview of the experimental setup of the life-history experiment (A, *Experiment I*) and the cellular response experiment (B, *Experiment II*). Both experiments included three pre-exposure host densities: high-density (striped bar), low-density (white bar) and simulated high-density (grey bar), and *D. magna* were fed four different food levels 0.25, 0.5, 1.0 and 2.0 absorbances (abs; one abs is the optical absorbance of 650 nm white light by the algae culture). On the day that *D. magna* reached maturity (between day 8–12) they were exposed to the bacterial parasite *P. ramosa* for 5 hours. Each jar had its own day of maturity. On day 35 post exposure (p.e.) all infected *Daphnia* were sacrificed and transmission spores were counted in A, while day X indicates the day the last healthy *Daphnia* had died and life history measures were terminated.

This experiment was terminated on day 35 post-exposure. Surviving hosts were frozen individually in a 1.5 ml Eppendorf tube for spore counting. *Daphnia* that died prior to termination of the experiment were frozen for spore counting purposes on the day of death. Parasite spore number was taken as a measure of parasite fitness, and was determined as follows: the host body was crushed in 500 µl de-ionised water using a plastic Pellet Pestle; this solution was then vortexed and spores were counted using a CASY Cell Counter (Model TT) according to the manufacturer’s protocol. Subsequently, the number of transmission spores per *Daphnia* was calculated from the respective dilutions. For a schematic drawing of the experimental protocol see Fig. 1.

### Experiment II

Five hours after parasite exposure, all five *Daphnia* of each replicate were placed in a Petri dish and the *Daphnia* hearts were pierced with a 25-guage needle (BD Microlance, Drogheda, Ireland). From each individual 0.5 µl of haemolymph was taken up with a 10 µl TipOne Repel Polymer Technology pipette tip (StarLab, Ahrensburg, Germany), pooled per replicate and mixed with 4 µl of ice-cold anticoagulant buffer (98 mM NaOH, 186 mM NaCl, 17 mM EDTA and 41 mM citric acid, pH adjusted to 4.5, [32]). Of this suspension, 4 µl were placed in a fertility-counting chamber 0.001 mm^2^×0.100 mm, Hawksley, Lancing, Sussex, UK), and the number of haemocytes was counted. For a schematic drawing of the experimental protocol see Fig. 1B.

### Statistical Analyses, Experiment I

We first examined if juvenile density or food availability, or both affected the number of offspring in the first clutch and the age at first reproduction. As exposure to the parasite only occurred once the first clutch was laid in the brood chamber, these first clutch traits cannot have been influenced by the parasite. This analysis thus allowed us to detect if our pre-exposure food and density treatments were generally effective and impacted *Daphnia* life history. Size of the first clutch was analysed with a univariate general linear model (GLM), and age at first reproduction, being a time to event variable, was analysed with Cox regression (proportional hazards). Finally we studied the proportion of infected hosts and how infection was influenced by density and food with a generalized linear model (GLM) with binomial error distribution.

To determine how our treatments (juvenile density and food) affected the performance of infected hosts relative to uninfected hosts, we added infection status (two levels, healthy or infected) as an explanatory variable to the model that also included density and food level. Thus, it was the interactions between infection status and food and density levels that were the main explanatory variables of interest. We analysed the following response variables: number of offspring, host survival and parasite transmission spore production. The number of offspring and parasite transmission spores production were ln-transformed and studied with a univariate GLM. Time to host death and time to castration (measured as the day that offspring production ceased due to parasite infection) were analysed using a Cox-regression (proportional hazards). For the survival data we censored our data with 1 being individuals that were dead and 0 individuals still alive when last seen. The number of offspring per *Daphnia* did not include the first clutch, as it was unlikely to have been affected by the parasite treatment because of the timing of parasite exposure. All analyses were performed in SPSS 19.

### Statistical Analyses, Experiment II

Haemocyte counts were square-root transformed and subjected to a univariate GLM with the dependent variable “haemocytes per µl” and the independent variables density, food level and parasite exposure (exposed or unexposed). All analyses were performed in SPSS.

## Results

### Experiment I

**Figure 2:**
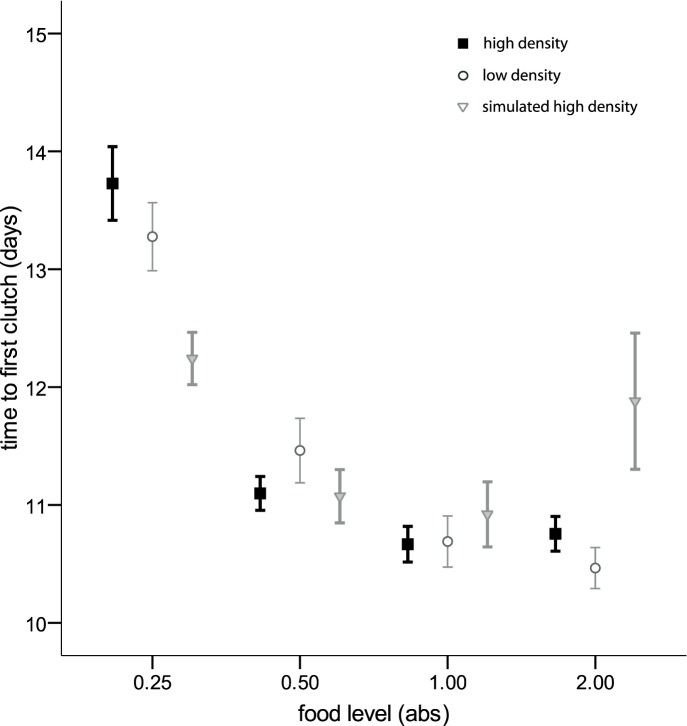
Time to first reproduction in days (mean +/− SE) depicted for all *D. magna* in relation to four different food levels 0.25, 0.5, 1.0 and 2.0 absorbances (abs; one abs is the optical absorbance of 650 nm white light by the algae culture). Note that this trait is not yet influenced by parasite infection. Black symbolizes the high-density treatment, light grey the low-density treatment, and dark grey the simulated high-density treatment.

**Figure 3:**
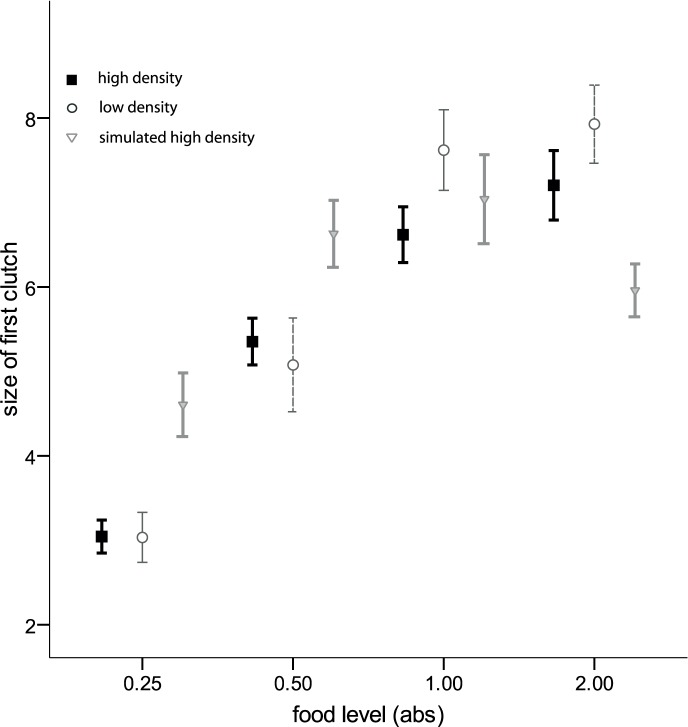
Size of first clutch (mean +/− SE) depicted for all *D. magna* in relation to four different food levels 0.25, 0.5, 1.0 and 2.0 absorbances (abs; one abs is the optical absorbance of 650 nm white light by the algae culture). Note that this trait is not yet influenced by parasite infection. Black symbolizes the high-density treatment, light grey the low-density treatment, and dark grey the simulated high-density treatment.

*Daphnia* life history was significantly affected by both juvenile food and density treatments. There was also a food by density interaction for both these response variables (size of first clutch: F_6, 384_ = 4.82, p<0.001; age at first reproduction: N = 408, Wald-Chi^2^ = 20.59, p = 0.002) and higher food levels led to earlier age at first reproduction (N = 408, Wald-Chi^2^ = 9.78, p = 0.020, Fig. 2) and larger first clutches (F_3, 384_ = 55.47, p<0.001, Fig. 3); juvenile density only affected the age at first reproduction (N = 408, Wald-Chi^2^ = 13.42, p = 0.001, Fig. 2).

The mean prevalence of infection was 85% with prevalences ranging from 94.3% (0.5 abs food) to 76.1% for (1.0 abs). However, this was not significantly affected by the pre-exposure food (F_3, 197_ = 2.393, p = 0.070) or density treatments (F_2, 197_ = 1.003, p = 0.369). Time to castration took 10.9 days ±0.25 (SE) and was independent of both food treatment (Wald-Chi^2^ = 2.19, p = 0.534) and host juvenile density (Wald-Chi^2^ = 0.492, p = 0.782). Specifically, 50% of hosts were castrated by day 10, and only 2.4% of infected hosts had more than 3 clutches before castration. Uninfected hosts had 6.9±0.06 (SE) clutches.

**Figure 4:**
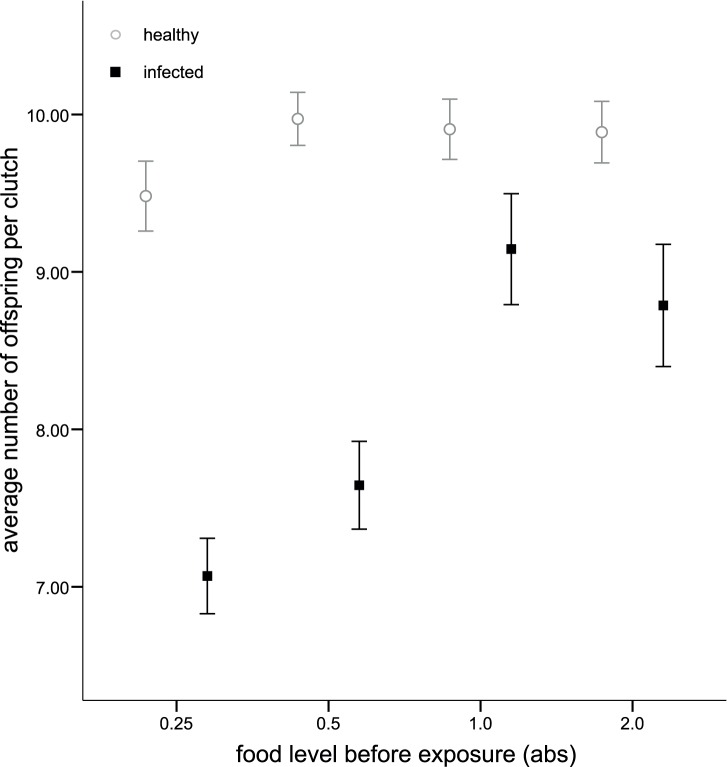
Number of offspring per clutch in *P. ramosa* infected and healthy *D. magna* hosts in relation to four different food levels 0.25, 0.5, 1.0 and 2.0 absorbances (abs; one abs is the optical absorbance of 650 nm white light by the algae culture; mean +/− SE). First clutch was removed from analysis since it was deposited before parasite exposure and thus does not influence the cost of infection. Black depicts *P. ramosa* infected animals, and grey uninfected *D. magna*. See Table 1 for statistical details.

Unexposed hosts had 65.07±11.2 (SE) offspring, whereas infected individuals produced only 21.13±6.0 (SE). However, we were most interested in how pre-exposure food or density modified consequences of infection, which would be evident as an infection status by treatment interaction with one of the response variables. Such interactions were not apparent for parasite transmission spore production or host mortality, but an interaction between infection status and juvenile food level was observed for total host fecundity (Table 1); infected hosts had fewer offspring in each clutch they had (Fig. 4), but this was modified by food such that infected hosts receiving the higher pre-exposure food levels had clutch sizes that approached those of uninfected hosts.

**Table 1:** Effects of *P. ramosa* infection (healthy/infected), food (four different levels) and density (three levels) on *D. magna* fecundity, parasite transmission spores measured per infected *D. magna* as well as host survival measured for parasite-exposed animal.

	df	F or Wald-Chi^2^	p
**Parasite transmission spores (GLM)**			
food	3	0.487	0.692
density	2	1.399	0.253
food × density	6	0.233	0.964
**Time to host death (Cox)**			
infection	1	20.183	**<0.001**
infection × density	2	0.088	0.957
infection × food	3	1.003	0.801
food	2	0.622	0.733
density	2	1.339	0.512
infection × food × density	6	3.567	0.312
density × food	6	3.806	0.703
**Total host fecundity (GLM)**			
infection	1	1928.8	**<0.001**
food	3	16.04	**<0.001**
density	2	0.284	0.753
infection × density	2	0.201	0.818
infection × food	3	3.90	**0.009**
infection × food × density	6	1.608	0.144
**Number of offspring per clutch (GLM)**			
infection	1	1027.48	**<0.001**
food	3	0.960	0.412
density	2	0.360	0.698
infection × density	2	0.367	0.693
infection × food	3	4.36	**0.005**
infection × food × density	6	0.396	0.881

The test statistic is either an *F*-ratio (using a GLM) or a Wald-Chi^2^ (using a Cox regression). P is the level of significance, df the degrees of freedom. Significant p-values are highlighted in bold.

### Experiment II

**Figure 5:**
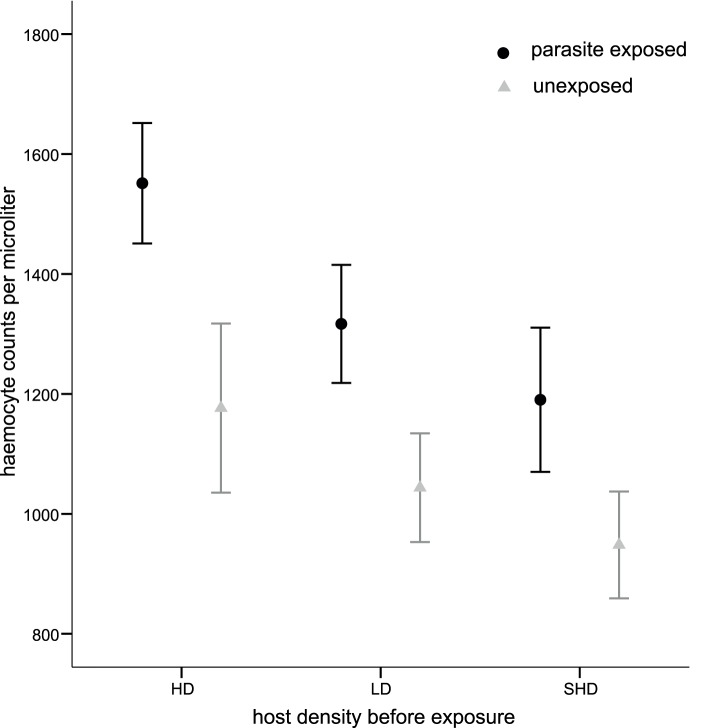
Number of circulating haemocytes of *D. magna* adult hosts reared at three different juvenile densities: high-density (HD), low-density (LD) and simulated high-density (SHD); average haemocyte counts per µl. Black depicts *P. ramosa* exposed individuals and grey unexposed *D. magna* (mean +/− SE).

Parasite-exposed *Daphnia* had more circulating haemocytes than controls (N = 238, F_1, 214_ = 10.44, p = 0.001). The pre-exposure density treatments affected haemocyte counts, with the number of haemocytes being highest in *Daphnia* that experienced high juvenile host density (HD) previous to parasite exposure (F_2, 214_ = 3.88, p = 0.022, Fig. 5). However, variation in pre-exposure food treatment did not affect haemocyte numbers (F_3, 214_ = 2.03, p = 0.111).

## Discussion

Environmental heterogeneity, such as variation in population density and food levels, may affect the expression of infection-related traits and thus alter host and parasite fitness [33], [34]. The present study analysed the effects of varying juvenile host densities and food availability on *D. magna* fitness under infection with the sterilizing parasite *P. ramosa*. This study is among the first to experimentally test for the host density dependence of virulence, which has been proposed by recent theoretical models [5], [6], [35], and to disentangle pre-exposure host density from pre-exposure food availability.

Food treatment had a large impact on host fitness: juvenile *Daphnia* from the lowest food treatment produced 24% fewer offspring than those from the highest food treatment. However, neither the food nor the density treatments affected the probability of becoming infected. As expected, the infected hosts were eventually sterilised by the parasite. Even prior to complete sterilisation, infected hosts showed reduced fecundity. This reduction in fecundity was dependent on juvenile food treatment: the parasite-induced reduction in fecundity was large in the low food environment, but when food was abundant, infected and healthy *Daphnia* differed only slightly in their early reproduction (Fig. 4). It is important to note that sterilisation has the biggest effect on host fitness. However, small changes in numbers of offspring in early clutches could have large effects on population size in the future, given that early population growth is exponential. A decline in food availability could thus reduce the supply of susceptible hosts, lower the incidence of infection and potentially terminate epidemics. This is in line with results by Vale et al. [30] who studied the environmentally mediated tolerance to infection in the same host-parasite system. Vale et al. [30] observed a much more benign parasitic interaction under high food conditions, whereas under food-limited conditions, the parasite severely damaged its host. Whilst well-fed mothers will suffer less virulence, the offspring may in fact suffer more. Specifically, well-fed *D. magna* tend to produce smaller, lower-quality offspring that are also relatively susceptible to parasites, compared to offspring of mothers in poor condition [36]–[38]. And yet these offspring of well-fed mothers may reproduce more in the absence of parasites [39]. The population dynamic and epidemiological consequences of these interactions between maternal food, current food, reproduction and susceptibility (or virulence) remain to be determined.

We estimated parasite fitness by measuring the production of transmission spores within infected hosts. Unlike host fecundity, parasite transmission spore production was not affected by the food treatment experienced by juvenile hosts. These results suggest that past low food conditions increase virulence without affecting parasite transmission potential (Fig. 4). These results were surprising, as low food for hosts should reduce host reproduction as well as parasite reproduction since both are using the same food source. Based on past studies [30], [31], we expected parasite fitness to be linked to host quality, and in particular that there would be many more spores produced under high food conditions. Both Ebert et al. [31] as well as Vale et al. [30] continued with food treatments after parasite exposure and collected parasite transmission spores on the day the hosts died. Hence, they collected spores from *Daphnia* that died “naturally”, i.e. once host death was induced by the parasite. In contrast, we stopped our food and density regimes upon parasite exposure (as we had to sacrifice all individuals from Experiment II at this point) and collected parasite spores at a fixed point in time, prior to host death. Past studies have shown that the number of spores produced will increase with time, but show similar patterns with respect to treatment effects [40]. This is why we presumed that it was not strictly necessary for hosts to die before counting transmission spores. However, it remains conceivable that methodological differences between studies account for these discrepancies.

In *D. magna*, the cellular response is thought to occur as *Pasteuria* transmission spores pass from the gut to the haemocoel; it is a consequence of infection rather than a cause of resistance [28]. We found juvenile *Daphnia* kept at high-density had the highest baseline (pre-exposure) haemocyte counts (Fig. 5). However, these high haemocyte counts were not associated with increased prevalence of infection in parasite-exposed hosts. The lack of statistical interaction between the juvenile density treatments and parasite exposure on haemocyte number shows that the juvenile density treatments neither strengthen nor weaken the host’s cellular response to parasite exposure. Still, we need to keep in mind that haemocytes are also involved in key physiological processes other than immunity [41], [42]: higher baseline haemocyte numbers may thus reflect other physiological stresses associated with crowding. Testing very low densities (<5 *Daphnia*) while keeping food constant could help to disentangle the relationship between density and immunity for *D. magna*. A follow-up study could test haemocyte expression upon exposure to a parasite other than *Pasteuria* and to a non-pathogenic immune stimulant. Such an experiment could detect if there is a general immune system mechanism for which costs are expected to be high in a parasite free environment.

Simulated high-density (SHD) was included to study differences between actual, physical crowding and perceived crowding (which is likely mediated through chemical cues of conspecific individuals dissolved in the water). While the host’s cellular immune response significantly differed between SHD and HD, this was not in the direction we expected. Overall, the interpretation of the SHD results is not straightforward; for example, time to first reproduction under SHD (Fig. 2) was, compared to the other two juvenile host density treatments, shorter in low food conditions but longer in high food conditions. For the size of the first clutch (Fig. 3) we found the opposite: clutches under SHD were larger in low food conditions but smaller than those of the other density treatments when food level was high. Therefore, the reasons for the outcome of our experiment might be more complex than we expected, and SHD might not solely cue for crowded conditions. It could also signal anoxia and/or contain cues from degenerating algae, or there could be unknown ecological or physiological interactions between food level and crowding, including processes that we did not control for and of which we do not know the exact effects on *Daphnia* fitness.

Overall, the effect of pre-exposure host density was rather weak compared to other studies investigating host density without parasite exposure [43], [44]. We did not detect a significant effect of juvenile host density (before parasite exposure) on host fecundity. However, unlike previous studies, we were able to disentangle juvenile host density from food availability. As we only found direct effects of food, our findings suggest that density-dependent effects act mainly through correlated effects on food availability. For the snail *P. antipodarum,* Neiman et al. [43] showed that negative-density dependence is mainly caused by food limitation, while Burns [44] detected depressed growth and lower reproduction in small-bodied *Daphnia* under crowded conditions. As in the present work, both studies observed complex effects between food level, host density, and host fitness. While the high and low host densities (15 and 5 individuals per 200 ml) we used lay within the range of a natural population [45], even higher host densities might be required to cause sufficient stress lasting over a long enough period in order to detect its responses in *Daphnia* life-history traits. Generally, there remains a lack of empirical studies combining (juvenile) food availability, (juvenile) host density and parasitism, and our results indicate how such multi-factorial interactions are much more complex than generally expected. To disentangle the effects of food availability, host density and parasite exposure we intentionally kept the genetic component constant for this study (one host clone and one parasite strain), but future experiments might profit from a higher number of host clones and parasite strains.
